# Editorial: Antimicrobial Resistance in Aquatic Environments

**DOI:** 10.3389/fmicb.2022.866268

**Published:** 2022-02-24

**Authors:** William Calero-Cáceres, Elisabet Marti, Jorge Olivares-Pacheco, Lorena Rodriguez-Rubio

**Affiliations:** ^1^UTA-RAM-One Health, Department of Food and Biotechnology Science and Engineering, Universidad Técnica de Ambato, Ambato, Ecuador; ^2^Department of Food Microbial Systems, Agroscope, Bern, Switzerland; ^3^Facultad de Ciencias, Grupo de Resistencia Antimicrobiana en Bacterias Patógenas y Ambientales GRABPA, Instituto de Biología, Pontificia Universidad Católica de Valparaíso, Valparaiso, Chile; ^4^Department of Genetics, Microbiology and Statistics, University of Barcelona, Barcelona, Spain

**Keywords:** antimicrobial resistance, aquatic environment, One Health, sewage, rivers/streams, ARGs antibiotic resistance genes

The current SARS-CoV-2 pandemic has exacerbated the rapid diagnosis of infectious diseases outbreaks to take suitable epidemiological measures to minimize negative impacts (Oude Munnink et al., 2021). However, the silent pandemic of antimicrobial resistance (AMR) faces several outstanding questions about its evolution and dissemination. The urgency of an integrated approach involving all ecological compartments, where antimicrobials and antimicrobial resistance genes (ARGs) reservoirs are generated, maintained, and disseminated, is urgently required (Da Silva et al., 2020). Aquatic environments are critical for understanding how the AMR develops and spreads worldwide, considering their role as an endpoint of effluents of wastewater treatment plants (WWTPs) or direct disposition of sewage from human or animal origin (Zheng et al., 2021; Miłobedzka et al., 2022), the runoff of biosolids in the agriculture (Buta et al., 2021), and other anthropogenic factors that contribute to the propagation of antimicrobial resistance determinants.

Therefore, this Research Topic aimed to deliver state-of-the-art knowledge and ideas on aquatic environments' role in selecting, maintaining, and dispersing AMR determinants. Fourteen articles from Europe, Asia, America, and Africa have been published on this topic that complements our knowledge and formulate several questions for the scientific community worldwide.

## Hospital Wastewater Role on AMR Dissemination

Hospital wastewaters represent a broad reservoir of antibiotic-resistant bacteria (ARB) and ARGs, which include extended-spectrum β-lactamases (ESBLs) and carbapenemase-producing *Enterobacteriaceae* (CPE), for instance (Hassoun-Kheir et al., 2020). However, there are significant knowledge gaps about the proper wastewater treatment technologies to be applied and a lack of protocols and indicators for executing an appropriate risk assessment (Nguyen et al., 2021). This Research Topic includes articles that cover the influence of hospital discharges on their receiving water bodies. In Romania, the study of Popa et al. describes the transmission of multidrug-resistant *Klebsiella pneumoniae* ST101 clone from hospital to wastewater and its persistence after chlorine treatment. The article highlights the risk of inappropriate hospital sewage disposition onto the surface water and their potential implications on the trophic chain. In Brazil, the study of Esposito et al. reports the genomic data and the virulence potential of *Pseudomonas aeruginosa* that harbor the São-Paulo-Metallo-β-lactamase (SPM-1), carried by high-risk clone ST277 isolated from urban rivers. The authors observed a common resistome and virulome between clinical and environmental SPM-1-producing *P. aeruginosa* strains endemic from Brazil. Additionally, the SNP-based phylogenomics showed a high similarity between clinical and environmental genomes, suggesting that these clones could be disseminated onto water bodies from hospital settings.

## Metagenomics as a Promising Tool for AMR Surveillance in the Environment

One of the current outstanding questions about the analysis of AMR in the environment is the standardization of genomic and metagenomic assays that could minimize the spatiotemporal variability, the allochthonous ARG levels, the environmental resistome complexity, and the biases about genomic extraction, sequencing, and genomic analyses (Calero-Cáceres et al., 2019; Li et al., 2020). Two articles that highlight the advantages of metagenomics in AMR analysis were included in this Research Topic. First, Perry et al. analyzed the influence of different hospital clinical activities on the abundance of ARGs in hospital wastewater in Scotland, highlighting the advantages of shotgun metagenomics to identify a full range of ARGs that could be used to guide environmental policies about AMR. Additionally, the article of Guo et al., using a high-throughput sequencing-based metagenomic approach, investigated the composition of bacteria and ARGs in wastewater from hospitals in China, suggesting a correlation between the abundance of ARGs and specific bacterial genera and remarking that it is necessary to complement their study including physicochemical analysis for the raw wastewater. Both articles show interesting results and note the necessity to develop an integrative framework that would include omics, physicochemical and epidemiological research to enhance the evaluation of ARGs pollution in environmental sources.

## Urban WWTP Influence on AMR Dissemination

Several papers of this special issue analyzed the influence of WWTPs and their discharges on AMR dissemination: In South Korea, Shin et al. characterized an extensively drug-resistant (XDR) *E. coli* isolated from influents of a WWTP. This study suggests that these isolates could be disseminated into the outgoing river from WWTP. The sewage could act as a potential spreader of ARGs, including emerging carbapenemase genes like *bla*_NDM−5_. In South Africa, Mbanga et al. characterized isolates of *Enterococcus* spp. from a WWTP and their receiving water bodies that serve as a water source for domestic, agricultural, and recreational purposes. Those isolates harbor a wide plethora of ARGs and virulence factors, showing that the effluents of the WWTP could act as a dissemination vector of multidrug-resistant (MDR) microorganisms. This Research Topic includes a review paper by Uluseker et al. that extensively reviews the current knowledge on sources, spread, and removal mechanisms of ARGs in microbial communities of wastewaters, WWTPs, and downstream recipients. This review includes the basis of antibiotic resistance, an explanation about the dynamics of AMR and antibiotics in WWTPs, and suggestions to be considered for the operation, regulation, and design of WWTPs. These studies suggest the urgent need for regular surveillance and management of water bodies to limit the spread of these isolates.

## Anthropogenic Influence on Water Bodies

Singh et al. analyzed *Escherichia coli* from the river Yamuna (India), a highly polluted river that receives an intense anthropogenic influence from urban and animal origin. Their results showed high AMR profiles, highlighting the presence of CTX-M-15 type ESBLs and the occurrence of class I integrons in their isolates. In Ireland, Sala-Comorera et al. demonstrated the strong impact of different watercourses discharges onto the levels of AMR in both bacterial and bacteriophage fractions in marine bathing waters, which may expose the users to fecal pollution and therefore could increase the probability to be exposed to ARGs. Another outstanding question about AMR in the environment is to demonstrate which levels of AMR are necessary to represent a real environmental danger. Finally, Pallares-Vega et al., shown by *in vitro* assays the role of ecological factors that could hamper conjugative plasmid transfer from gut bacteria once discharged into the environment. Their findings highlight the possibility that the fecal organisms may transfer plasmids in aquatic ecosystems, despite the variable conditions that could occur environmentally.

## AMR Impact on the Food Chain

The food supply chain connects environmental sources of bacteria with humans and represents another outstanding field in the One Health perspective for understanding the dissemination and evolution of AMR. The article of Montero et al. analyzed ESBL producing *E. coli* isolated from irrigation waters, vegetables, and fruits in Ecuador. These authors detected that the allelic variants of the *bla*_CTX−*M*_ gene found in irrigation channels and vegetables were the same as those observed in commensal *E. coli* from domestic animals, and commensal and pathogenic *E. coli* from humans, suggesting a connection between these different sources. In addition, the article of Cheng et al. analyzed sediments from aquaculture farms in China by constructing network plots based on *16S rRNA* metagenomics, physicochemical analysis, and quantification of ARGs. Their results provide evidence for understanding the environmental risks associated with aquaculture practices. On the other hand, Ye et al. showed in *Edwardsiella tarda*, an important pathogen in aquaculture, that reactive oxygen species (ROS) play a role in bacterial resistance and sensitivity to ceftazidime. They saw a lower ROS production in ceftazidime-resistant *E. tarda* than in a sensitive strain related to the inactivation of the pyruvate cycle. Additionally, their study reveals a new mechanism that increases ROS production, the activation of the pyruvate cycle by Fe^3+^. These findings provide tools and knowledge for future new strategies to fight MDR pathogens.

## Role of Wildlife as a Potential Reservoir of AMR

Zeballos-Gross et al. comprehensively reviewed the potential role of gulls as reservoirs and vectors of AMR in the environment, highlighting the current knowledge about related research, the phenotypic and molecular characterization of AMR, limitations about the existing methodologies, and suggestions for improving the derived results.

In summary, this Research Topic provides an excellent update of the role of aquatic ecosystems on the evolution and dissemination of AMR worldwide. Therefore, the editors encourage the scientific community to consider the results and challenges of this special issue.

## Author Contributions

WC-C, JO-P, LR-R, and EM contributed to analyzing the reviewed articles and preparing the manuscript. All authors contributed to the article and approved the submitted version.

## Funding

LR-R is a Serra Húnter Lecturer.

## Conflict of Interest

The authors declare that the research was conducted in the absence of any commercial or financial relationships that could be construed as a potential conflict of interest.

## Publisher's Note

All claims expressed in this article are solely those of the authors and do not necessarily represent those of their affiliated organizations, or those of the publisher, the editors and the reviewers. Any product that may be evaluated in this article, or claim that may be made by its manufacturer, is not guaranteed or endorsed by the publisher.
